# Intracellular Ca^2+^ Concentration and Phosphatidylserine Exposure in Healthy Human Erythrocytes in Dependence on *in vivo* Cell Age

**DOI:** 10.3389/fphys.2019.01629

**Published:** 2020-01-10

**Authors:** Ingolf Bernhardt, Duc Bach Nguyen, Mauro C. Wesseling, Lars Kaestner

**Affiliations:** ^1^Laboratory of Biophysics, Faculty of Natural Science and Technology, Saarland University, Saarbrücken, Germany; ^2^Experimental Physics, Faculty of Natural Science and Technology, Saarland University, Saarbrücken, Germany; ^3^Theoretical Medicine and Biosciences, Medical Faculty, Saarland University, Homburg, Germany

**Keywords:** red blood cells, aging, Ca^2+^ content, phosphatidylserine exposure, lysophosphatidic acid, flow cytometry

## Abstract

After about 120 days of circulation in the blood stream, erythrocytes are cleared by macrophages in the spleen and the liver. The “eat me” signal of this event is thought to be the translocation of phosphatidylserine from the inner to the outer membrane leaflet due to activation of the scramblase, while the flippase is inactivated. Both processes are triggered by an increased intracellular Ca^2+^ concentration. Although this is not the only mechanism involved in erythrocyte clearance, in this minireview, we focus on the following questions: Is the intracellular-free Ca^2+^ concentration and hence phosphatidylserine exposure dependent on the erythrocyte age, i.e. is the Ca^2+^ concentration, progressively raising during the erythrocyte aging *in vivo*? Can putative differences in intracellular Ca^2+^ and exposure of phosphatidylserine to the outer membrane leaflet be measured in age separated cell populations? Literature research revealed less than dozen of such publications with vastly contradicting results for the Ca^2+^ concentrations but consistency for a lack of change for the phosphatidylserine exposure. Additionally, we performed reanalysis of published data resulting in an ostensive illustration of the situation described above. Relating these results to erythrocyte physiology and biochemistry, we can conclude that the variation of the intracellular free Ca^2+^ concentration is limited with 10 μM as the upper level of the concentration. Furthermore, we propose the hypothesis that variations in measured Ca^2+^ concentrations may to a large extent depend on the experimental conditions applied but reflect a putatively changed Ca^2+^ susceptibility of erythrocytes in dependence of *in vivo* cell age.

## Introduction

The formation of erythrocytes named erythropoiesis takes about 7 days (Silbernagel and Despopoulus, 2007). It happens before birth in the yolk sac, liver, spleen, and bone marrow and after birth only in the red marrow of the plates and short bones (Dzierzak and Philipsen, 2013). From multipotent stem cells, erythroblasts first emerge, which still have a nucleus. After erythroblasts have lost their nucleus and organelles, they are called reticulocytes and migrate into the blood stream. The normal lifespan of an erythrocyte is about 120 days. One of the first data analysis giving a result of about 120 days was published by Callender et al. (1945).

In a drop of human blood one can obviously find a composition of cells in all different ages. There is an age-dependent variation in their density. Young cells have a significantly lower density in comparison to old cells (Piomelli and Seaman, 1993). Based on this, it is possible to separate the erythrocytes into at least five fractions by density gradient centrifugation making use of, e.g., stractan (Ballas et al., 1986; Waugh et al., 1992) or Percoll (Lutz et al., 1992; Makhro et al., 2016). Although there are exceptions (Lew and Tiffert, 2013), this difference in density makes it possible to investigate cell physiological parameters of cell populations in dependence on the age of the cells.

It has been reported that physiological erythrocyte aging results in a decreased cell volume, size, and mean corpuscular volume (Nash and Wyard, 1980; Linderkamp and Meiselman, 1982; van Oss, 1982; Bosch et al., 1992). In addition, the 2,3-diphosphoglycerate/hemoglobin ratio (Samaja et al., 1990) and the deformability (Clark et al., 1983; Gifford et al., 2006) are reduced in aged erythrocytes. Further parameters are increased, such as mean corpuscular hemoglobin concentration (Bosch et al., 1992), glycated hemoglobin (Bunn et al., 1976), osmotic fragility (Rifkind et al., 1983), and creatine levels (Syllm-Rapoport et al., 1981). Seppi et al. (1991) showed that band 3 protein and membrane skeleton proteins undergo conformational changes and/or oxidation with increasing cell age. This was confirmed by Castellana et al. (1992). In contrast, Piccinini et al. (1995) reported that the oxidation state of membrane proteins does not seem to change during the erythrocyte life span. A decrease of the anti-oxidant defense-related activity when erythrocytes increase their age was reported by D’Alessandro et al. (2013). Ciana et al. (2004) described an increased Tyr-phosphorylation level of band 3 protein of old erythrocytes compared to younger ones under hypertonic conditions. Furthermore, it is well known that there is an age-dependent increase of the protein ratio of band 4.1a:4.1b proteins, which is regarded as a molecular clock. Measurements of the 4.1a:4.1b ratio make it possible to determine differences in cell age of erythrocytes separated in fractions (Mueller et al., 1987; Inaba and Maede, 1988; Kaestner and Minetti, 2017).

The factors, crucial for the aging process and the mechanisms for the removal of damaged or old erythrocytes from the circulation are not yet fully understood. However, once erythrocytes reach a threshold for intracellular free Ca^2+^, the associated phosphatidylserine exposure leads to erythrocyte clearance (Kuypers and de Jong, 2004; Bosman et al., 2005; Bogdanova et al., 2013; de Back et al., 2014). However, it is unclear if the way to the increased Ca^2+^ concentration is a continuous process accompanying the erythrocyte aging or a rather rapid process directly triggering the removal.

Here, we provide the context and a literature review about reports investigating if there is an *in vivo* age dependence of the intracellular Ca^2+^ concentration and phosphatidylserine exposure in healthy human erythrocytes. The focus on these parameters is by no means a statement that this process is regarded as the most important in erythrocyte clearance. We focus on healthy human erythrocytes and refer to primarily *in vivo* conditions, explicitly excluding processes occurring during erythrocyte storage in particular storage lesions (Flatt et al., 2014; Petkova-Kirova et al., 2018).

## The Intracellular Ca^2+^ Concentration, Phosphatidylserine Exposure, and Their Dependence of Erythrocyte Age

Historically, first investigations described an increase of the total intracellular Ca^2+^ concentration in the process of human erythrocyte aging. La Celle et al. (1973) reported an increase from 60 μM in young erythrocytes to 100 μM in old ones, whereas Shiga et al. (1985) found only an increase from 15 to 33 μM Ca^2+^. However, physiologically important is the free cytosolic Ca^2+^ concentration, which is the portion of Ca^2+^ freely available to the cytosolic and membrane proteins (Tiffert et al., 2002), i.e., the portion of Ca^2+^ acting as messenger in cellular signaling. In all following statements, the Ca^2+^ concentration refers to the free intracellular Ca^2+^ concentration.

The intracellular Ca^2+^ concentration of erythrocytes can be determined under physiological conditions by different methods such as Ca^2+^ chelators and atomic absorption spectroscopy. Fluorescent indicators for Ca^2+^ such as Fura-2, Fluo-3, and Fluo-4 have been commonly applied (for Fura-2 see, e.g., Romero et al., 1997). However, Kaestner et al. (2006) pointed out that the application of Fura-2 is problematic in human erythrocytes because its excitation and emission properties are distorted by the absorption of hemoglobin. Additionally, ultraviolet light as required for the excitation of Fura-2 may photo-convert hemoglobin into fluorescent photoproducts (Kaestner et al., 2006). Therefore, the quantitative measurement of the free intracellular Ca^2+^ concentration of individual erythrocytes is not possible with the ratiometric Ca^2+^ fluorophores Fura-2 or Indo-1 (Kaestner et al., 2006). However, it appears reasonable to determine the average physiological intracellular Ca^2+^ concentration in human erythrocytes to be below 100 nM (Tiffert et al., 1993; Tiffert and Lew, 1997). Although the single-cell measurements based on the fluorescent dyes Fluo-3 and Fluo-4 do not allow a quantitative measurement of the Ca^2+^ concentration (Kaestner et al., 2006), they reveal a high intercellular variation (Wang et al., 2014). A cell is typically regarded to show an increased intracellular Ca^2+^ concentration, when fluorescence intensity increase exceeds three times the standard deviation of the fluorescence recorded during control conditions (Wang et al., 2013).

The in principle low intracellular Ca^2+^ concentration represents the balance between the passive Ca^2+^ influx and the active Ca^2+^ extrusion (Ca^2+^ efflux) realized by the Ca^2+^ pump. The passive Ca^2+^ influx is mediated through low capacity transport pathways with carrier properties (Ferreira and Lew, 1977; Desai et al., 1991), ion channels (Kaestner et al., 2020) and a putative “leak.” Interestingly, Lew et al. (2007) found that the plasma membrane Ca^2+^ pump activity declines with erythrocyte age. In a variety of reports, we have described possible mechanisms leading to an increased intracellular Ca^2+^ content of erythrocytes, mainly based on the activity of ion channels (Kaestner et al., 2000; Kaestner and Bernhardt, 2002; Wagner-Britz et al., 2013; Fermo et al., 2017; Danielczok et al., 2017; Rotordam et al., 2019; for a recent review see Kaestner et al., 2020).

Increase in free intracellular Ca^2+^ activates the lipid scramblase (see e.g. Woon et al., 1999; Lang et al., 2004, 2005; Nguyen et al., 2011). This lipid translocator in turn mediates a significant exposure of phosphatidylserine on the outer membrane leaflet (Woon et al., 1999). At the same time the flippase, which actively (ATP-dependent) transports phophatidylserine from the outer membrane leaflet to the inner one is inhibited (Devaux et al., 2008). Although the flippase shows an almost complete inactivation at a Ca^2+^ concentration of 400 nM (Bitbol et al., 1987), for physiological Ca^2+^ concentrations, total suppression of flippase activity leaves the membrane asymmetry undisturbed (Arashiki et al., 2016). In contrast, for scramblase, the values for half maximal activation were determined by different studies with varying methodologies and slightly different results. Values varied between approximately 30 μM determined in liposomes (Stout et al., 1989) and 70 μM measured in erythrocyte ghosts (Woon et al., 1999). Considering the Hill coefficient describing the steepness of the slope for the activation and the predominant role of the scramblase shown by the application of the specific scramblase inhibitor R5421 (Wesseling et al., 2016a), an enhanced free Ca^2+^ content above 10 μM in erythrocytes will result in an increase in phosphatidylserine on the outer membrane leaflet (Bogdanova et al., 2013). An increase in phosphatidylserine of the outer membrane leaflet of the erythrocyte we regard as any Annexin-V-fluorophore signal above zero, practically this is regarded to be the case when the signal exceeds the average background value by two times the noise amplitude.

After this methodological and molecular-mechanistical view on the intracellular Ca^2+^ concentration and phosphatidylserine exposure, we want to take a closer look on the cell age dependence of these parameters. Therefore, we searched the literature for investigations of intracellular-free Ca^2+^ and phosphatidylserine exposure measurements but excluded *in vitro* and artificial aging and focused exclusively on healthy human erythrocytes.

Concerning the free intracellular Ca^2+^, it has been reported that older erythrocytes contain an increased free intracellular Ca^2+^ concentration (Aiken et al., 1992; Romero et al., 1997). Combining a fluorinated calcium chelator probe (5,5’-difluoroBAPTA) and fluorine magnetic resonance (^19^F-NMR) technique Aiken et al. (1992) found for the young and old cell fraction of erythrocytes a mean intracellular-free Ca^2+^concentration of 62 and 221 nM, respectively.

In contrast, for the very young red blood cells, the reticulocytes de Haro et al. (1985) reported an increased intracellular Ca^2+^ concentration compared with mature erythrocytes, whereas Wiley and Shaller (1977) measured that reticulocytes are more permeable to Ca^2+^ than mature cells but their intracellular Ca^2+^ concentration is not increased.

Wesseling et al. (2016b) reported a lack of difference in the intracellular Ca^2+^ concentration and in the phosphatidylserine exposure in dependence on erythrocyte age (based on density separated erythrocytes).

**Figure 1:**
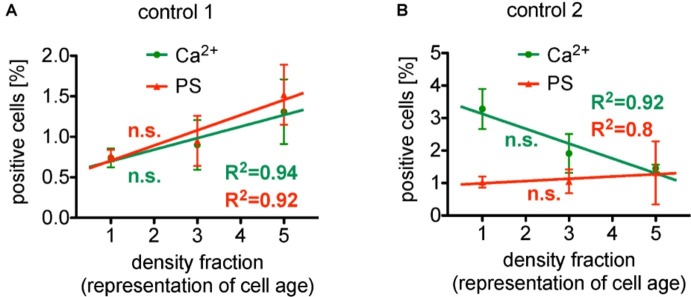
Reanalysis of data initially presented in Wesseling et al. (2016b). In the original publication, all fractions were only compared in pairs, while here we followed the approach to plot (and analyze) the measured effect in dependence of the cell age. Both panels present the situation under control conditions (without pharmalogical stimulation). **(A)** The percentage of erythrocytes showing increased Ca^2+^ content as well as phosphatidylserine exposure depicts a linear behavior in dependence of cell age with a very good regression, R^2^ given in the figure. However, the slope of these linear regressions failed to be significantly different from zero, i.e., failed to show a significant change. **(B)** The percentage of erythrocytes showing decreased Ca^2+^ content, while phosphatidylserine exposure depicts a linear increase in dependence of cell age, again, with a very good regression, R^2^ given in the figure. The slope of both linear regressions also failed to be significantly different from zero, i.e., failed to show a significant change. Furthermore, comparing the particular fractions between the two measurements **(A,B)** applying an unpaired *t*-test with Welch’s correction (unequal SD values), none of the fractions showed a significant difference (*p* > 0.05). Both measurements **(A,B)** were performed in the same laboratory. Blood samples were given from healthy sportsmen (Department of Sports Medicine) with an age between 18 and 36 years. Each data point consists of three donors (not identical between **A** and **B**) and for each donor and condition, 90,000 cells were analyzed by flow cytometry. The only obvious difference is the composition of the solutions. [**(A)** in mM]: 145 NaCl, 7.5 KCl, 2 CaCl_2_, 10 glucose, 10 HEPES, pH 7.4 (Tyrode solution); [**(B)** in mM]: 125 NaCl, 5 KCl, 1 CaCl_2_, 1 MgCl_2_, 5 glucose, 32 HEPES, pH 7.4 (Ringer solution). **(A)** A reprint from Bernhardt et al. (2019).

Taken all these reports together, we have a very controversial situation. To further illustrate this conflicting situation, we present a reanalysis of previously published data (Wesseling et al., 2016b). The percentage of erythrocytes showing an enhanced Ca^2+^ concentration as well as phosphatidylserine exposure present a linear relation relative to the density centrifugation fractions representing erythrocyte age. The regression values R^2^ for Ca^2+^ and phosphatidylserine of 0.94 and 0.92, respectively, speak for themselves (Figure 1A). Surprisingly, in a different set of experiments under conditions with similar characteristics (Figure 1B), the intracellular Ca^2+^ was linear decreasing (R^2^ of 0.92), while the phosphatidylserine exposure was increasing (R^2^ of 0.80). The only differences between the two separate measurements (A and B) were the donors (but all healthy) and the composition of the solutions (see legend of Figure 1). However, the major point is that the slope of the linear regressions in both conditions (A and B) failed to be significantly different from zero, i.e., albeit we have very nice (high R^2^) regression lines for different conditions, the data from Wesseling et al. (2016b) show no dependence of intracellular Ca^2+^ concentration and phosphatidylserine exposure from erythrocyte age. In other words: Although the panels of Figure 1 seem contradicting, from a statistical point of view they are consistent.

For phosphatidylserine exposure in erythrocytes, Franco et al. (2013) reported independence on cell age. Only after extended storage of erythrocytes for 2 days in Ringer solution (cp. Figure 1B), a significant enhancement in the intracellular Ca^2+^ concentration and also an increase in the phosphatidylserine translocation with cell age could be observed (Ghashghaeinia et al., 2012). The latter result was confirmed by Wesseling et al. (2016b). As this resembles storage conditions it cannot be regarded as reflecting *in vivo* conditions. In contrast, short-time (30 min) incubation experiments showed that there was neither significant difference of the Ca^2+^ content (see above) nor a phosphatidylserine translocation depending on cell age when comparing age populations in pairs (Wesseling et al., 2016b).

Summarizing this section, it is still elusive if there is an alteration of intracellular Ca^2+^ in dependence of erythrocyte age (several conflicting reports). However, since there is consistence in the reports concerning phosphatidylserine exposure (no change with cell age), we can conclude that the variation of the free intracellular Ca^2+^ concentration does not exceed 10 μM, which resembles the onset of the scramblase activation while the flippase is inactivated (Bogdanova et al., 2013).

## Stimulation of Ca^2+^ Entry and Increased Phosphatidylserine Exposure in Dependence on Erythrocyte Age

Pharmacologically, the Ca^2+^ entry into erythrocytes can be stimulated by lysophosphatidic acid or prostaglandin E_2_ (Li et al., 1996; Yang et al., 2000; Kaestner et al., 2004). Although the process itself is well known, the molecular signaling leading to the Ca^2+^ entry is still controversial (Yang et al., 2000; Kaestner et al., 2004; Wagner-Britz et al., 2013). Both substances (lysophosphatidic acid and prostaglandin E_2_) are released from activated platelets and therefore resemble a physiological stimulation occurring *in vivo* (in healthy humans). Under mechanical stress, prostaglandin E_2_ can even be released by the erythrocytes themselves (Oonishi et al., 1998). However, we were able to demonstrate that after activation of erythrocytes (e.g., by lysophosphatidic acid) most erythrocytes with increased Ca^2+^ content also responded with phosphatidylserine exposure (Nguyen et al., 2011).

**Figure 2:**
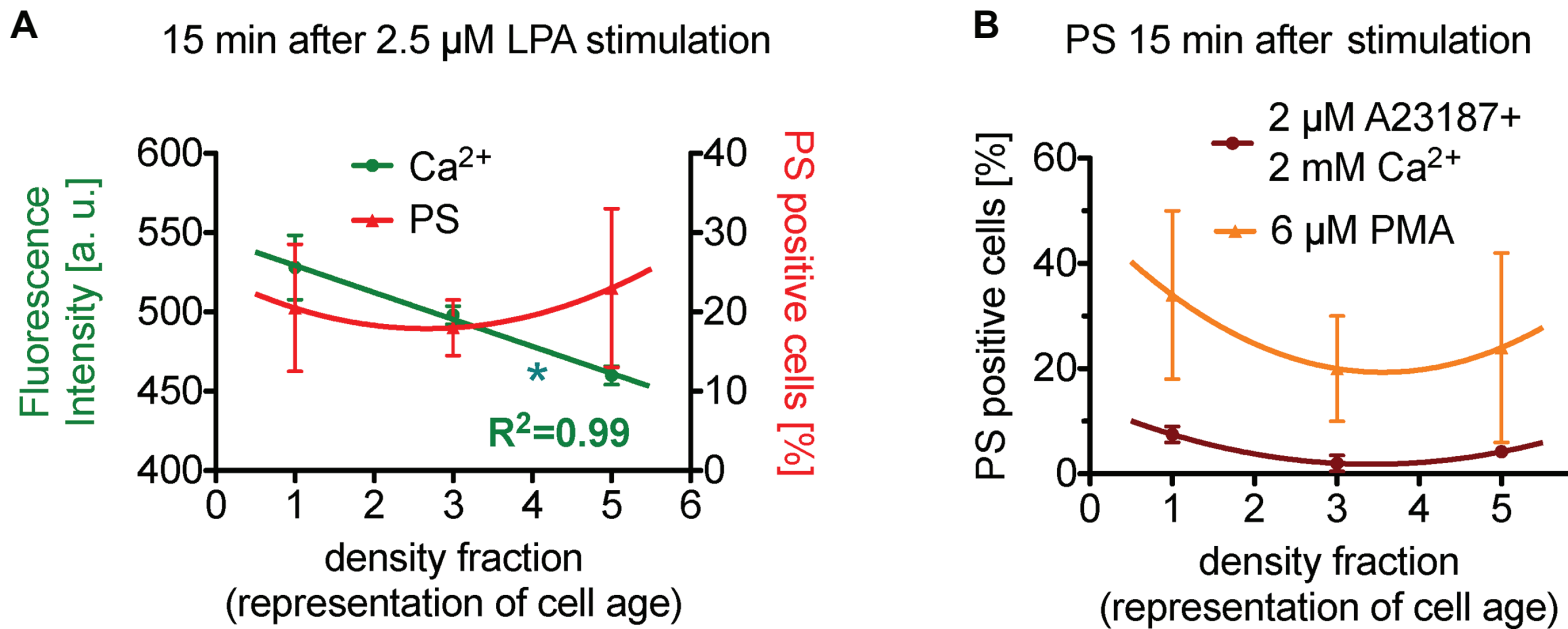
Reanalysis of data initially presented in Wesseling et al. (2016b). In the original publication, all fractions were only compared in pairs, while here we followed the approach to plot (and analyze) the measured effect in dependence of the cell age. **(A)** Presents the situation after 15 min of stimulation with lysophosphatidic acid (LPA). While the Ca^2+^ concentration seems to relate inversely proportional to erythrocyte age (slope is significant different from zero, *p* < 0.05 is marked with *), phosphatidylserine positive cells show a rather quadratic dependence on cell age. **(B)** Presents exclusively the phosphatidylserine exposure under more direct stimulations (15 min), namely the direct increase in intracellular Ca^2+^ in all cells by application of the Ca^2+^ ionophore A23187 in 2 mM Ca^2+^ containing solutions (dark red circles) and by a direct Ca^2+^-independent activation of protein kinase Cα (PKCα, orange triangles) by phorbol-12 myristate-13 acetate (PMA, an unspecific activator of conventional and novel PKCs but PKCα is the sole PKC of these 2 groups found in erythrocytes). Although different in amplitude, both stimulations result in a quadratic dependence on cell age. Blood samples were given from healthy sportsmen (Department of Sports Medicine) with an age between 18 and 36 years. Each data point consists of three donors and for each donor and condition 90,000 cells were analyzed by flow cytometry. For a complete dataset on the different stimulation modes, see Wesseling et al. (2016b). **(A)** A reprint from Bernhardt et al. (2019).

There is a debate in the literature whether erythrocytes depict a cell age dependency in the response of lysophosphatidic acid stimulated (see also above). To this end, we present a reanalysis of data by Wesseling et al. (2016b) as a plot depicting age dependence. After stimulation of the erythrocytes with lysophosphatidic acid, the cellular behavior is quite complex. Figure 2A shows the response after 15 min of 2.5 μM lysophosphatidic acid stimulation. While the Ca^2+^ content relate inversely proportional to erythrocyte age (slope significantly different from zero), phosphatidylserine positive erythrocytes depict a rather quadratic dependence on cell age.

Interestingly, in reticulocytes lysophosphatidic acid could not activate any measurable Ca^2+^ signals (Wang et al., 2013). To judge the quadratic age dependence of the phosphatidylserine exposure, we reanalyzed a more direct stimulation also published by Wesseling et al. (2016b). Figure 2B shows the percentage of phosphatidylserine exposing cells upon massive Ca^2+^ increase in all erythrocytes by use of the Ca^2+^ ionophore A23187 (dark red circles in Figure 2B) and by stimulation of protein kinase Cα (orange triangles in Figure 2B) by phorbol-12 myristate-13 acetate (for details, see Figure 2). Although different in amplitude, both stimulations also result in a quadratic response relative to the erythrocyte age (meaning the medium fraction always displays the lowest number in phosphatidylserine positive erythrocytes). For a thorough discussion of the relationship between intracellular Ca^2+^ increase and phosphatidylserine exposure upon phorbol-12 myristate-13 acetate stimulation of erythrocytes, we refer to Nguyen et al. (2011) and Bernhardt et al. (2019).

Although we have no mechanistic hypothesis for this quadratic response, the consistency along the various types of physiological [lysophosphatidic acid; concentrations of 1–5 μM when platelets are activated (Eichholtz et al., 1993)] and artificial (Ca^2+^ ionophore, phorbol-12 myristate-13 acetate) stimulation indicates such a dependence as a general property.

## Interpretations, Conclusions, and Outlook

We cannot solve the question whether the Ca^2+^ content of human erythrocytes is related to cellular aging at all. Nevertheless, intracellular Ca^2+^ is very likely be increased immediately prior erythrocyte clearance but since phosphatidylserine exposing erythrocytes are quickly cleared (Kuypers and de Jong, 2004; Bosman et al., 2005; Bogdanova et al., 2013; de Back et al., 2014) it is experimentally challenging to detect/measure the cells with increased phosphatidylserine exposure (see below for detailed discussion).

An initial explanation for varying and contradicting results might be caused by the measurement technique: While microscopy examines erythrocytes settled on a coverslip in a measurement chamber, the cells do not experience a serious mechanical challenge and therefore microscopy can be regarded as a rather gentle technique. In contrast, in flow cytometry measurements, the erythrocytes experience severe mechanical forces, like high pressure and significant shear forces (Minetti et al., 2013). As a result, a decent number of erythrocytes with enhanced Ca^2+^, which are more fragile, simply may lyse when passing the flow cytometer. This means the number of cells with increased intracellular Ca^2+^ might by systematically decreased in flow cytometry experiments. The variety in specifications of flow cytometers may even add to the heterogeneity in measurements throughout different laboratories.

Another more conceptual interpretation is based on the above described findings that (1) increase in the intracellular Ca^2+^ concentration and phosphatidylserine exposure of erythrocytes increases with storage time in physiological (Ca^2+^ containing) solutions in an age-dependent manner (Ghashghaeinia et al., 2012) and (2) *in vivo*, phosphatidylserine exposing cells are cleared by macrophages in the liver and the spleen (Kuypers and de Jong, 2004; Bosman et al., 2005; de Back et al., 2014). Therefore, *in vivo,* these cells are filtered out, making putative age differences invisible. Older cells may have a higher susceptibility for Ca^2+^ (maybe just by a decreased Ca^2+^ pump activity) and not necessarily an increased Ca^2+^ concentration. The measurements *in vitro* may then reflect the experimental conditions addressing this susceptibility (cp. Figure 1), which is likely to be modulated by a plethora of conditions and parameters, such as type of anticoagulant, temperature and temperature changes, modulations in gravity (extend and time of centrifugation), pH value, mechanical stimulation (vortexing), composition of solution (salts, additives, solvents), and other metabolic conditions, just to name a few.

The Ca^2+^ concentration we measure might thus be the product of an (*in vivo*) susceptibility for Ca^2+^ and the (*in vitro*) experimental conditions. Our measurements may therefore reflect the erythrocyte susceptibility for Ca^2+^ if we manage to keep the experimental conditions constant, which can be achieved in a laboratory but makes direct comparison of independent studies of different laboratories almost impossible. Furthermore, there might be conditions (in addition to the absence of external Ca^2+^) that do not act on the susceptibility for Ca^2+^ (factor zero in the above-mentioned product).

This hypothesis has the potential to explain the greatly variable and partly contradicting experimental results concerning the dependence of intracellular Ca^2+^ in erythrocytes in dependence on cell age. Previous observations of highly variable Ca^2+^ concentrations after blood sample shipments (Hertz et al., 2017) support this hypothesis. However, further investigations are required to substantiate the hypothesis and continue to explore the phenomenon of the variability of intracellular Ca^2+^ measurements.

Finally, the conflicting reports reveal once more the need for intralaboratory and interlaboratory validation of protocols by quality controls in particular for the measurement of intracellular Ca^2+^ as we previously proposed for erythrocyte research in general (Minetti et al., 2013).

## Author Contributions

All authors listed have made a substantial, direct and intellectual contribution to the work, and approved it for publication.

### Conflict of Interest

The authors declare that the research was conducted in the absence of any commercial or financial relationships that could be construed as a potential conflict of interest.
